# Epicardial Ablation as a Treatment of Recurrent Ventricular Tachycardia Originating From a Left Ventricular Aneurysm: A Case Report and Review of the Literature

**DOI:** 10.7759/cureus.35509

**Published:** 2023-02-26

**Authors:** Ngoda Manongi, Jim Cheung, Seth Goldbarg

**Affiliations:** 1 Internal Medicine, NewYork-Presbyterian Queens, Flushing, USA; 2 Cardiac Electrophysiology, Weill Cornell Medical Center, New York, USA; 3 Cardiac Electrophysiology, NewYork-Presbyterian Queens, Queens, USA

**Keywords:** non-valvular atrial fibrillation, left ventricular apical aneurysm, arrhythmogenic, epicardial ablation, radiofrequency ablation (rfa), ventricular tachycardia (vt)

## Abstract

Catheter ablation (CA) is an important therapeutic modality for the management of ventricular tachycardia (VT). In some patients, CA may be ineffective because of the inability to reach the effective target site from the endocardial surface. Partly, this is due to the effect of the transmural extent of the myocardial scars. The operator’s ability to map and ablate the epicardial surface has enhanced our understanding of scar-related VT in various substrate states. A left ventricular aneurysm (LVA) that develops after myocardial infarction may increase the risk of VT. Endocardial ablation alone of LVA may be insufficient in preventing recurrent VT. Numerous studies have demonstrated greater freedom from recurrence with adjunctive epicardial mapping and ablation via a percutaneous subxiphoid technique. Currently, epicardial ablation is performed predominantly at high-volume tertiary referral centers via the percutaneous subxiphoid approach. In this review, we first report a case of a man in his 70s with ischemic cardiomyopathy, a large apical aneurysm, and recurrent VT status post-endocardial ablation who presented with incessant VT. The patient underwent successful epicardial ablation over the apical aneurysm. Second, our case showcases the percutaneous approach and underscores its clinical indications and potential complications.

## Introduction

Ventricular arrhythmias including ventricular tachycardia (VT) are responsible for hundreds of thousands of deaths annually [1]. VT can be defined as an arrhythmia originating from the ventricles with a rate that exceeds 100 beats per minute (bpm), usually associated with a wide QRS complex ≥120 ms. VT can occur in the absence or presence of structural heart disease. In patients with structural heart disease, the most common substrate for re-entrant VT is scar tissue from previous myocardial infarction (MI) [2]. Most cardiomyopathies are predisposed to developing VT and include, among others, arrhythmogenic right ventricular cardiomyopathy, dilated cardiomyopathy, and hypertrophic cardiomyopathy [3]. In addition, left ventricular aneurysms (LVAs) and pseudoaneurysms are two complications of MI that can lead to serious morbidity and even death [4]. LVA formation involves the dilation of the ventricular wall that leads to a well-delineated outward bulging of the affected LV wall due to myocardial thinning and scar formation. LVA that develops after myocardial infarction may lead to VT, among other complications including thrombus formation [4]. LVAs themselves are potential arrhythmogenic substrates and, given their complex and often apical anatomy, may be difficult to target from the endocardium [4].

Catheter ablation (CA) of VT is an essential component of the management of VT. However, in many patients, despite medical and interventional therapy, VT recurs [3]. Almost three decades since its introduction in the mid-1990s, epicardial ablation has become a feasible technique for the treatment of VT in different cardiomyopathies after Sosa et al. described it for the treatment of the epicardial VT substrate in Chagas disease [5]. Since then, this technique has become an integral modality in the treatment of VT in high-volume clinical centers [6]. However, there continues to be a scarcity of data regarding the safety, efficacy, and clinical outcomes of CA of VT, particularly in patients with LVA.

## Case presentation

**Figure 1:**
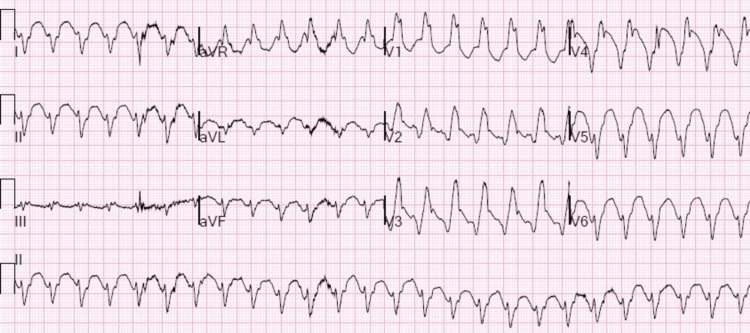
Electrocardiogram demonstrating sustained monomorphic ventricular tachycardia with a ventricular rate of 155 beats per minute.

A man in his 70s with paroxysmal atrial fibrillation and recurrent VT status post-endocardial ablations twice for VT (the last two months prior to admission), ischemic cardiomyopathy with a large apical/septal aneurysm, and implantable cardioverter-defibrillator (ICD) presented for evaluation of chest pain and recurrent palpitations. His recent ablation had targeted the endocardial border zone of the aneurysm. The patient stated that he had experienced palpitations for a week before he came to the hospital. He had been evaluated by his cardiologist three days prior to the presentation due to ICD therapy after ongoing palpitations for over an hour. His cardiologist increased amiodarone from 200 mg daily to 400 mg twice a day but the patient experienced recurrent palpitations associated with generalized weakness and dizziness. In the emergency department (ED), the patient was found to have a wide complex tachycardia and hypoxia requiring oxygen support. Blood work was significant for negative cardiac enzymes and mildly elevated N-terminal pro-b-type natriuretic peptide. The electrocardiogram (ECG) showed monomorphic VT at 155 bpm (Figure 1), with a right bundle branch block pattern and right superior axis consistent with LV origin. ICD interrogation revealed normal ICD function with VT below the detection limit of the device. The patient was given amiodarone 150 mg intravenous (IV) push and started on an amiodarone drip for 24 hours. He underwent successful defibrillation once with post-ECG showing sinus bradycardia at 45 with TWI in I, II, III, aVF, and V4-V6. The patient was admitted to the cardiac intensive care unit for close monitoring. He remained hemodynamically stable; however, given the patient’s history of recurrent VT, and the recent endocardial ablation, the decision was made to transfer him to a neighboring tertiary high-volume center to undergo epicardial access VT ablation.

**Figure 2:**
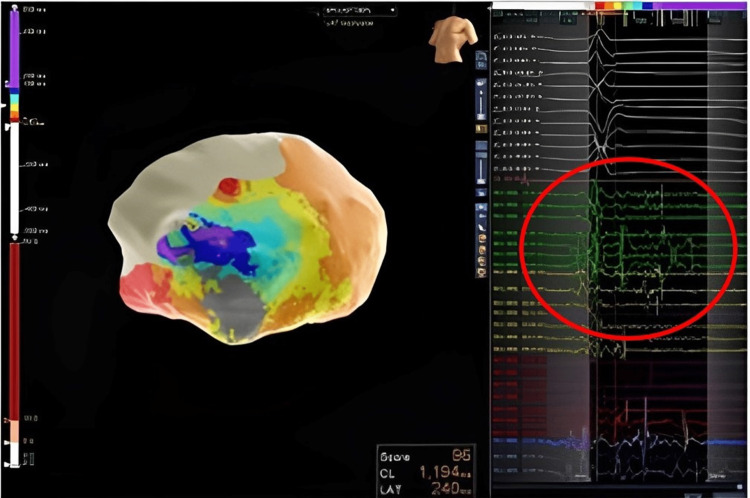
Epicardial mapping demonstrates late potentials (highlighted within the red circle) during sinus rhythm, indicative of areas of slow conduction overlying the aneurysm.

**Figure 3:**
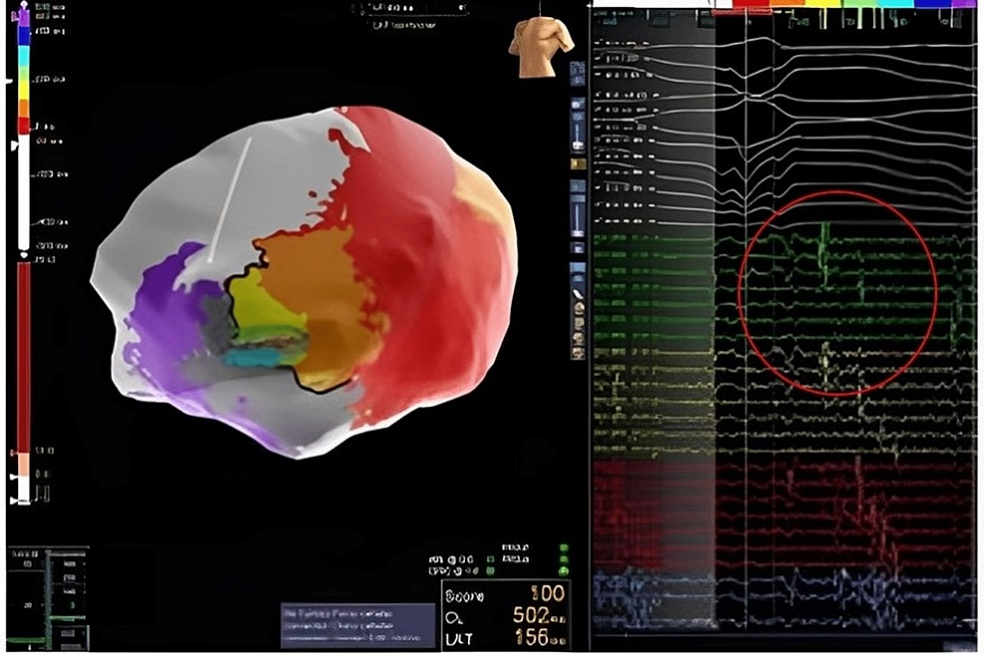
High-definition grid multipolar catheter signals at the critical isthmus demonstrating fractionated and late potentials (highlighted within the red circle) during ventricular tachycardia.

**Figure 4:**
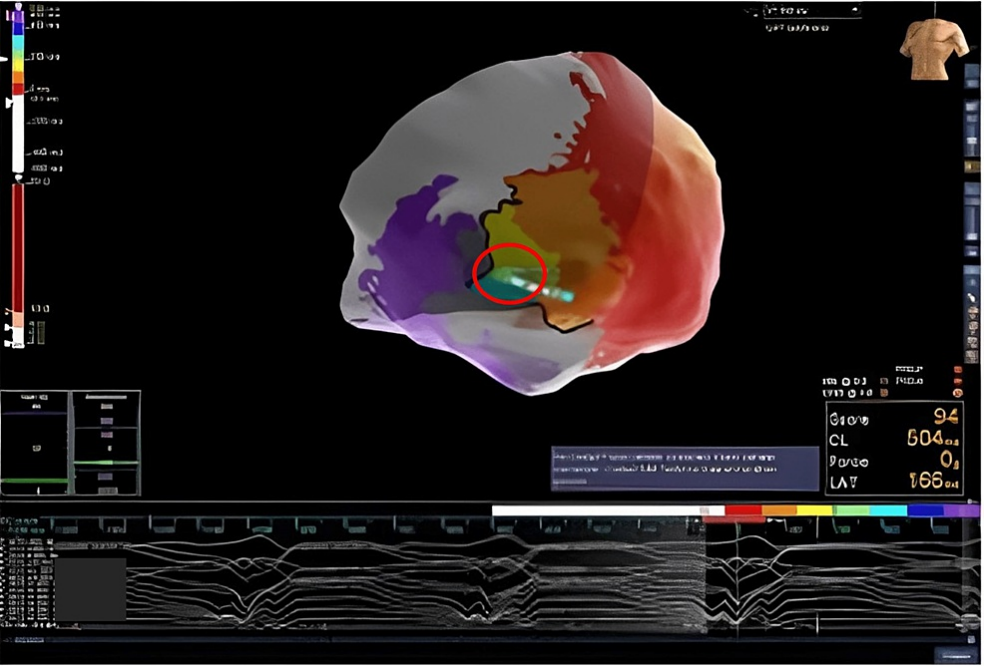
Ablation of a site (highlighted within the red circle) leading to ventricular tachycardia termination.

The procedure was performed under monitored anesthesia care via a femoral venous transseptal approach. The NavX system was used for electroanatomical mapping. Intracardiac ultrasound demonstrated no pericardial effusion and no thrombus in the apical aneurysm. The clinical VT was easily induced with a tachycardia cycle length (TCL) of 430 ms but due to hypotension was pace terminated. Epicardial access was obtained with a Tuohy needle and anterior subxiphoid approach under fluoroscopic guidance. Electroanatomic mapping demonstrated extensive scarring over the aneurysm but otherwise healthy voltage (Figure 2). Pace mapping demonstrated a 97% match to the clinical VT with a stim-QRS time of 114 ms, consistent with a possible exit site. A second VT was induced during pace mapping, showing a TCL of 520 ms, left bundle branch block superior axis, and V6 precordial transition, suggestive of a septal origin of the tachycardia and possible global myocardial involvement beyond the location of the aneurysm. This VT was hemodynamically stable and mapped with the HD grid (Figure 3). Activation mapping revealed a figure of eight VT with an isthmus at the anteroapical border zone and a septal exit (Figure 2). Entrainment from the perceived isthmus was concealed with post-pacing interval (PPI) - TCL = 0, with stim to QRS of 236 ms. VT1 was thought to share the same central isthmus with a more lateral exit site. Ablation at this site led to the termination of the VT in 14 seconds (Figure 4). Broad ablation at this border zone was performed. After the ablation, programmed stimulation with two drivetrains, and up to triple extra stimuli failed to induce sustained VT. The procedure was successful without any complications.

## Discussion

CA is often the most effective treatment for drug-refractory or incessant VT [7]. VT has been shown to be a marker for increased risk for heart failure and mortality [8,9]. While ICDs may prevent arrhythmia-related death by the termination of VT with either pacing or defibrillation, they do not reduce the future risk of ventricular arrhythmias and their downstream effects [9].

In ischemic scar-related VT, the most common substrate arises from the subendocardial segments of the myocardium [10]. For such patients, VT can be successfully ablated from the endocardium of the left or right ventricle via a transvenous transseptal or retrograde arterial approach [10]. However, substrates for VT are not limited to the subendocardial myocardium. Moreover, an LVA can become an arrhythmogenic substrate for LVA-related VT, which is usually located in the aneurysm border zone [11]. However, the characteristically thin and hyaline fibrous tissue of LVA’s apical aneurysmal wall makes the ablation of VT in patients with ischemic cardiomyopathy quite challenging [11]. The percutaneous epicardial approach for mapping and the accompanying ablation has been appropriately used for the treatment of such subepicardial VT [12]. The usual approach is subxiphoid epicardial access guided by fluoroscopy. Several factors need to be considered before utilizing the epicardial approach in VT ablation, such as a prior history of unsuccessful endocardial VT ablation, ECG criteria, substrate localization by imaging studies, the likelihood of epicardial VT in the underlying disease state, and intra-procedural mapping [12].

Epicardial ablation is most commonly employed after failed endocardial ablation [13,14]. This is because more than two-thirds of patients selected for epicardial mapping after failed ablation had an epicardial VT target [14]. Although not described in this case report, MRI and ECG criteria may be clinically useful when assessing for the presence of epicardial substrate. The use of epicardial ablation as a preemptive and adjunctive strategy has been reported in multiple observational studies [14-16]. Although the data on the safety and efficacy of CA for VT in patients with LVA is limited, the available studies indicate that ablation of VT in the presence of LVA can achieve relatively high efficacy and acceptable complication rates in high-risk patients [11,16].

The techniques employed during epicardial mapping are similar to when endocardial mapping is performed. However, a more careful examination of the regional anatomy prior to delivering radiofrequency energy on the epicardial surface needs consideration for potential collateral damage. This is because an 18-gauge introducer needle is needed to access the intra-pericadial space through subxiphoidal pericardial puncture, which can then proceed either anteriorly or posteriorly depending on the targeted substrate [6]. This approach is not benign. Several serious complications including left phrenic nerve damage, injury to the epicardial coronary arteries, hemopericardium, and pericarditis can occur as a result of thermal injury [17]. The most common complication is pericarditis, which is not surprising given the nature of the procedure, and operators sometimes instill intra-pericardial steroids at the end of the procedure to reduce the incidence of pericarditis [18,19]. Coronary arterial damage with epicardial radiofrequency ablation has been shown only in an animal model [20]. Real-time integration of CT imaging-derived coronary anatomy with electroanatomic mapping may be useful.

## Conclusions

In our case, we present a case of a man in his 70s with ischemic cardiomyopathy and a large apical aneurysm, who presented in incessant VT. He had undergone a recent endocardial ablation. The patient underwent successful epicardial VT ablation with the termination of VT at the first ablation site. While epicardial VT ablation has some unique risks, it may be the only effective treatment for certain VT circuits.
